# Binding to PI(4,5)P_2_ is indispensable for secretion of B-cell clonogenic HIV-1 matrix protein p17 variants

**DOI:** 10.1016/j.jbc.2021.100934

**Published:** 2021-07-15

**Authors:** Antonella Bugatti, Francesca Caccuri, Federica Filippini, Cosetta Ravelli, Arnaldo Caruso

**Affiliations:** 1 Section of Microbiology, Department of Molecular and Translational Medicine, University of Brescia Medical School, Brescia, Italy; 2 Section of Experimental Oncology and Immunology, Department of Molecular and Translational Medicine, University of Brescia Medical School, Brescia, Italy

**Keywords:** HIV, lymphoma, heparan sulfate, protein secretion, cell growth, BSA, bovine serum albumin, cART, combined antiretroviral therapy, DAPI, 4′,6-diamidino,2-phenylindole, EC, endothelial cell, EGM, endothelial cell growth medium, FBS, fetal bovine serum, HBR, highly basic region, HSPG, heparan sulfate proteoglycan, HUVEC, human umbilical vein endothelial cell, NHL, non-Hodgkin's lymphoma, p17, HIV-1 matrix protein p17, (PI(4,5)P_2_), phosphatidylinositol-(4,5)-bisphosphate, Pr55^*Gag*^, precursor Gag polyprotein, SPR, surface plasmon resonance, vp17, HIV-1 matrix protein p17 variant

## Abstract

HIV-1 matrix protein p17 variants (vp17s) derived from non-Hodgkin's lymphoma (NHL) tissues of HIV-1–seropositive (HIV^+^) patients promote B-cell growth by activating the Akt signaling pathway. It is fundamental to understand the role played by vp17s in producing a microenvironment that fosters lymphoma development and progression. Therefore, we asked whether vp17s could be secreted from infected cells in their biologically active form. In this study, we show that two B-cell growth-promoting vp17s, NHL-a101 and NHL-a102, characterized by amino acid insertions at position 117 to 118 (Ala–Ala) or 125 to 126 (Gly–Asn), respectively, are secreted from HIV-1–infected Jurkat T cells during the active phase of viral replication. Secretion of biologically active vp17s also occurred in HeLa cells nucleofected with a plasmid expressing the entire *Gag* gene, following proteolytic cleavage of the *Gag* precursor polyprotein (Pr55^*Gag*^) by cellular aspartyl proteases. Binding of Pr55^*Gag*^ to phosphatidylinositol-(4,5)-bisphosphate was indispensable for allowing the unconventional secretion of both wildtype p17 and vp17s. Indeed, here we demonstrate that inhibition of Pr55^*Gag*^ binding to phosphatidylinositol-(4,5)-bisphosphate by using neomycin, or its enzymatic depletion achieved by overexpression of 5ptaseIV, significantly impair the secretion of p17s. We also demonstrated that heparan sulfate proteoglycans were involved in tethering p17s at the cell surface. This finding opens up an interesting way for investigating whether tethered p17s on the surface of HIV-1 reservoirs may represent a likely target for immune-mediated killing.

The HIV-1 matrix protein p17 (p17) is a 132 amino acids–long *Gag*-encoded protein that plays a key role in virus assembly and release (1). Its interaction with many cellular proteins underlines the importance of the viral protein as a major determinant of human-specific adaptation (2). Virion-free p17 is released from HIV-1–infected cells, and it can be detected in blood at nanomolar concentrations (1, 3). Moreover, p17 accumulates and persists in different organs and tissues of patients even under successful combined antiretroviral therapy (cART) (4, 5, 6). In particular, a long-term p17 accumulation in lymph nodes of cART-treated patients has been shown, even after prolonged HIV-1 suppression (4). Taken together, these findings strongly suggest that p17 may be chronically present in the tissue microenvironment of HIV-1–seropositive (HIV^+^) patients, even during the pharmacological control of viral replication. These findings are consistent with several reports showing that the latently infected resting CD4^+^ T cells transcribe and translate *Gag* proteins, even without cell stimulation (7, 8), and that defective HIV-1 proviruses in the CD4^+^ T cells of HIV^+^ individuals on cART produce intact *Gag* proteins *in vitro* and *in vivo* (9).

Extracellularly, p17 has been found to deregulate the biological activity of different immune cells, which may be relevant in the context of viral pathogenesis. In particular, p17 is capable of triggering the activation, differentiation, and proliferation of different target immune cells as T cells (10, 11), natural killer cells (12), monocytes (13), and plasmacytoid dendritic cells (3). We also highlighted the capability of p17 to exert chemotactic activity on human primary B cells after binding to the C-X-C motif chemokine receptors 1 and 2 (14, 15). Interestingly, we showed that a p17 variant (vp17) carrying various amino acid substitutions, named S75X, derived from an Ugandan HIV-1 A1 strain, differently from a p17 derived from clade B virus (strain BH10; refp17), activated the Akt signaling pathway in B cells, thereby promoting cell growth and clonogenicity (16). This finding provided the first evidence on the existence of a vp17 with B-cell clonogenic activity on human B cells. More recently, in studies of p17 from human non-Hodgkin's lymphoma (NHL) cases, we characterized two categories of vp17s that promote B-cell growth and activate the Akt pathway: the first characterized by amino acid insertions at position 117 to 118 and the second characterized by amino acid insertions at position 125 to 126 (6). Ultradeep pyrosequencing showed that these two categories of vp17s are more frequently detected in plasma of HIV^+^ patients with NHL than without NHL (12). Moreover, we showed that vp17s characterized by an Ala–Ala insertion at position 117 to 118 (NHL-a101) and a Gly–Asn insertion at position 125 to 126 (NHL-a102), differently from refp17, were drastically destabilized. Because of misfolding, vp17s were found to expose a common functional epitope with B-cell clonogenic activity located at their N-terminal region (17). Recently, we demonstrated that the B-cell clonogenic activity of both NHL-a101 and NHL-a102 is mediated by activation of the protease-activated receptor 1, which triggers transactivation of the epidermal growth factor receptor (18). Collectively, our data support the hypothesis that certain vp17s may play a role in lymphoma pathogenesis. It is worth noting that in the cART era, B-cell lymphomas, and NHLs in particular, comprise more than 50% of all AIDS-defining cancers (19) and are still the most frequent cause of death in HIV^+^ patients (20), being often characterized by clinical aggressiveness (21, 22).

Despite the lack of signal sequence, similarly to other viral proteins (17, 23, 24, 25), p17 is secreted in a biologically active form by an unconventional pathway (26). Its secretion takes place at the plasma membrane of *Gag*-expressing cells, in the absence of active viral proteases. Secretion of p17 was found to be dependent on the interaction of *Gag* precursor polyprotein (Pr55^*Gag*^) with phosphatidylinositol-(4,5)-bisphosphate (PI(4,5)P_2_) and its subsequent cleavage from Pr55^*Gag*^ operated by cellular aspartyl proteases. We also highlighted that targeting Pr55^*Gag*^ to the plasma membrane through PI(4,5)P_2_ interactions is the only feasible mechanism for p17 secretion (26). Several groups have shown the role played by the highly basic region (HBR) located at the p17 N-terminal region in the p17 interaction with PI(4,5)P_2_ (27, 28, 29, 30). Moreover, some Pr55^*Gag*^ mutants in the HBR showed impaired Pr55^*Gag*^–PI(4,5)P_2_ interaction and relocalization of Pr55^*Gag*^ from the plasma membrane to intracellular compartments, thus impairing or abolishing p17 secretion (26). Because of their partial unfolding state and remodeling of their N-terminal region to expose the B-cell clonogenic epitope (17), we asked whether interaction of Pr55^*Gag*^ carrying vp17s with PI(4,5)P_2_ occurs, leading to Pr55^*Gag*^ cleavage by cellular or viral proteases and secretion as biologically active proteins. This is critical to hypothesize a role of vp17s in producing a microenvironment that fosters lymphoma development, progression, and metastasis.

Herein, we report that biologically active NHL-a101 and NHL-a102 vp17s are released by cells nucleofected with plasmid-expressing *Gag* or viral mutants. Finally, we show for the first time that secreted refp17 and vp17s are, at least in part, tethered to the cell surface through heparan sulfate proteoglycans (HSPGs).

## Results

### Quantification of vp17s secretion by T cells

**Figure 1:**
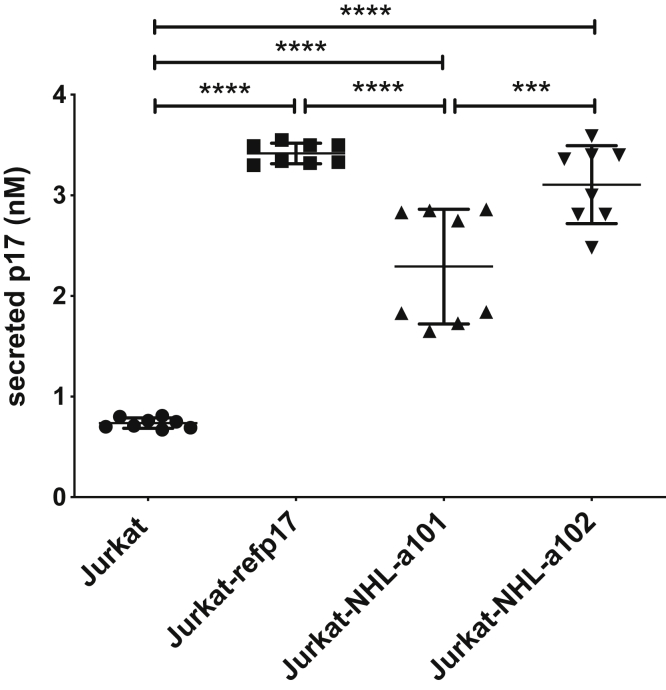
**Quantitative p17 secretion assay.** Evaluation of p17 release in the cell culture supernatant was performed by cellular ELISA. Jurkat cells were nucleofected with or without the pNL4-3 vector expressing refp17, NHL-a101, or NHL-a102 (Jurkat-refp17, Jurkat-NHL-a101, and Jurkat-NHL-a102, respectively). After 24 h, Jurkat were seeded onto ELISA plates precoated with anti-p17 mAb MBS-15 and incubated further for 24 h to allow protein secretion and accumulation. Quantification of released p17s was performed using a standard curve generated with recombinant proteins. Scatter plot was generated using the means of eight independent experiments. One-way ANOVA and the Bonferroni's post-test was used to compare data; ∗∗∗*p* < 0.001 and ∗∗∗∗*p* < 0.0001.

Our first aim was to assess if vp17s secretion occurs during active virus replication. Jurkat cells, which are similar to most HIV-1–infected CD4^+^ T cells (31), were transiently nucleofected with pNL4-3 plasmids expressing refp17, NHL-a101, or NHL-a102 (Jurkat-refp17, Jurkat-NHL-a101, and Jurkat-NHL-a102, respectively). Mock nucleofected cells were used as negative control (Jurkat). Twenty-four hours after nucleofection, 7.5 × 10^5^ cells/well were cultured for additional 24 h in complete medium in anti-p17 mAb MBS-15–coated wells of 96-well plates. Plate-bound p17, secreted by seeded Jurkat-nucleofected cells, was then quantified by ELISA (26). As previously reported, p17 was easily detected in the supernatant of Jurkat-refp17, and the amount of secreted p17 ranged from 3.30 to 3.55 nM (mean ± SD: 3.41 ± 0.10 nM). At the same time, vp17s were also secreted by Jurkat-NHL-a101 (from 1.65 to 2.86 nM, mean ± SD: 2.29 ± 0.57 nM) and Jurkat-NHL-a102 (2.48–3.59 nM, mean ± SD: 3.1 ± 0.39 nM) (Fig. 1). This result attests for the active secretion of refp17 and vp17s from infected cells.

### Secreted vp17s are biologically active

**Figure 2:**
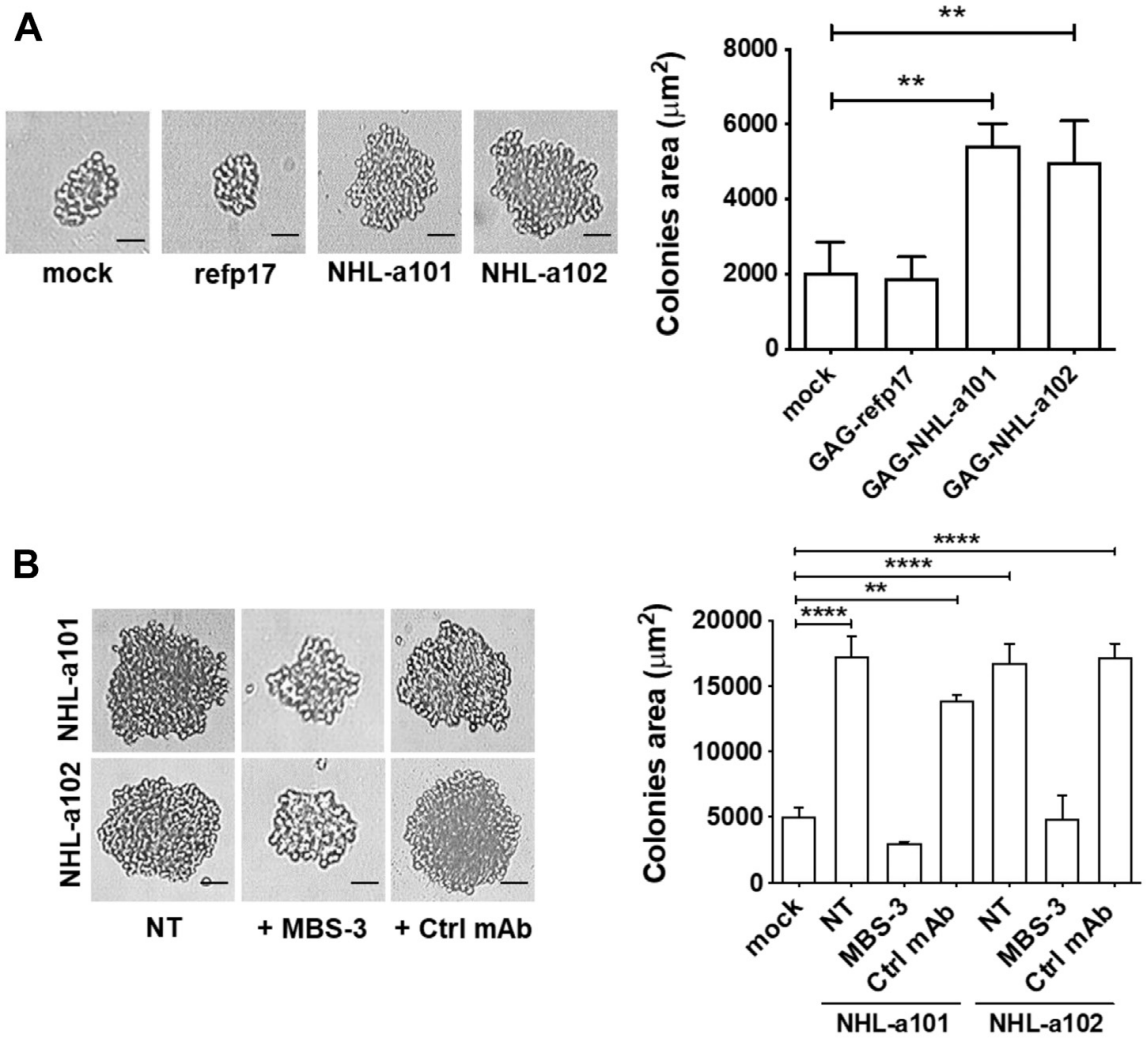
**Secreted vp17s induce B-cell growth and transformation.***A*, supernatants collected from HeLa cells nucleofected with *Gag*-expressing plasmids carrying refp17 (HeLa-refp17), NHL-a101 (HeLa-NHL-a101), or NHL-a102 (HeLa-NHL-a102) were used to resuspend Raji B cells (refp17, NHL-a101, and NHL-a102, respectively) and perform colony formation assay. Supernatant collected from mock nucleofected cells (mock) were used as negative control. *B*, for some experimental conditions, HeLa-conditioned supernatants were not treated (NT) or preincubated for 30 min at 37 °C with 1 μg/ml of unrelated Ctrl mAb or p17 neutralizing mAb MBS-3. Bright-field images represent the characteristic morphology of 2D colonies (*left panels*), one colony for each condition is shown (original magnification, 40×). The scale bar represents 25 μM. The colony area was measured (by using Leica Qwin image analysis software [*right panels*]). The graphs were generated using the means of three independent experiments. One-way ANOVA and the Bonferroni's post-test was used to compare data; ∗∗*p* < 0.01 and ∗∗∗∗*p* < 0.0001. vp17, HIV-1 matrix protein p17 variant.

We previously demonstrated that differently from refp17, NHL-a101 and NHL-a102 are able to induce B-cell growth and clonogenicity (6). To determine if vp17s are secreted in their biologically active form, we performed a single cell cloning assay on Raji B cells resuspended with supernatants collected from HeLa cells nucleofected with *Gag*-expressing plasmids carrying refp17 (HeLa-refp17), NHL-a101 (HeLa-NHL-a101), or NHL-a102 (HeLa-NHL-a102). As representatively shown in Figure 2*A*, a visible single colony was spontaneously developing in >60% of seeded wells at day 8 of culture, attesting for active cell proliferation. As expected, in the presence of vp17s released from nucleofected cells, colonies showed a significantly larger size than colonies cultured in medium of mock- or refp17-nucleofected cells.

To demonstrate that the observed B-cell growth and clone expansion was specifically mediated by vp17s, the B-cell cloning assay was performed in the presence or absence of the p17-neutralizing mAb MBS-3, which recognizes the clonogenic epitope of vp17s (17). An unrelated mAb (Ctrl mAb) was used as negative control in the assay. As representatively shown in Figure 2*B*, the presence of mAb MBS-3 (1 μg/ml) completely inhibited the clonogenic activity of both vp17s, whereas the Ctrl mAb (1 μg/ml) turned out to be ineffective.

**Figure 3:**
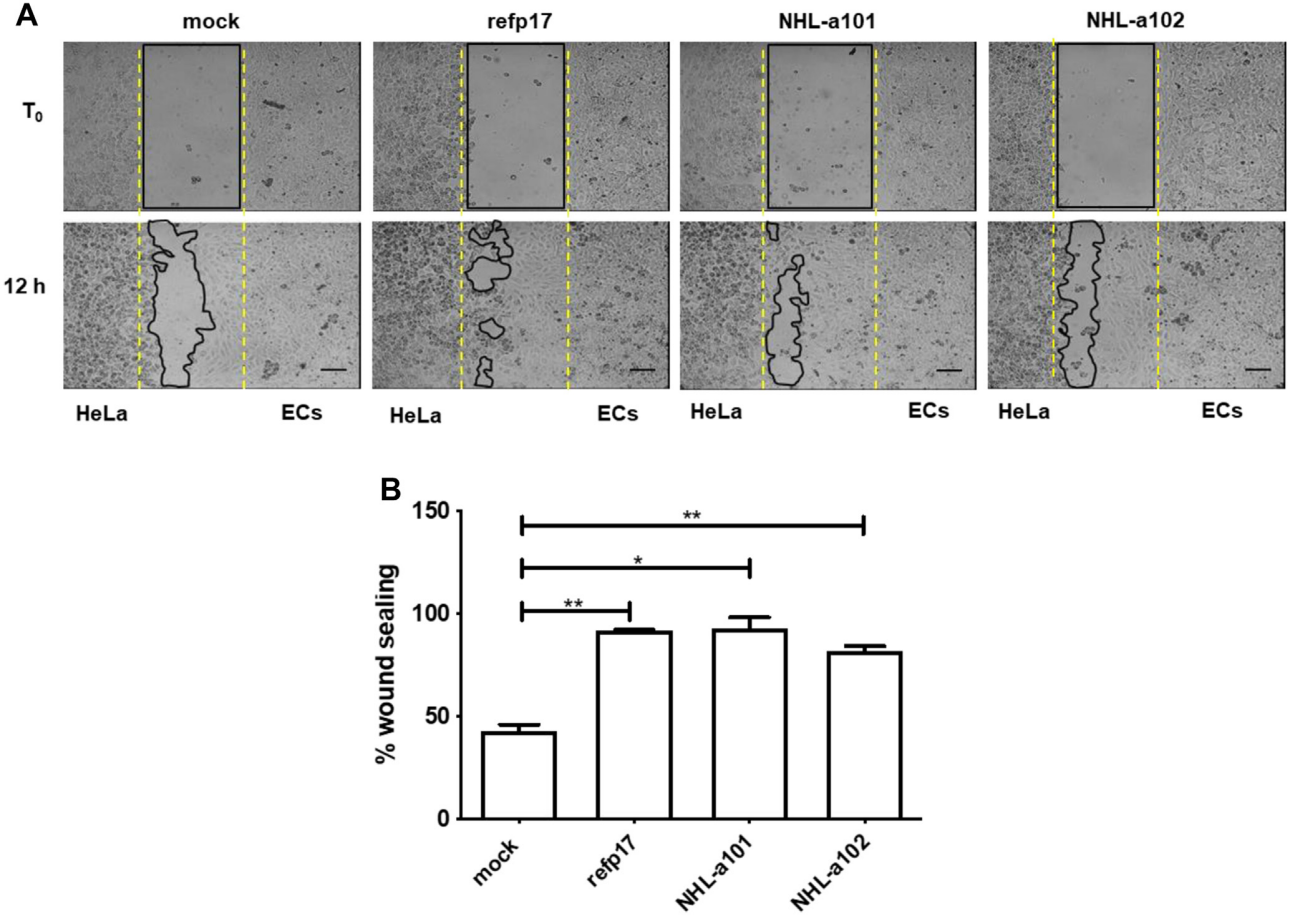
**Secreted p17s promote HUVEC migration.***A*, HeLa cells nucleofected with *Gag*-expressing plasmids carrying refp17 (HeLa-refp17), NHL-a101 (HeLa-NHL-a101), or NHL-a102 (HeLa-NHL-a102) were cocultivated with HUVECs in Culture-Insert 2 Well (refp17, NHL-a101, and NHL-a102, respectively). After 24 h of incubation, the insert in the plate was gently removed to create a scratch between HeLa and HUVEC (ECs) cells. Cell migration was recorded by light microscopy over a 12 h time course after wound scratch. Images are representative of three independent experiments with similar results (magnification, 10×). The scale bar represents 100 μm. *B*, graph represents quantitative analysis of wound sealing showing the percentage of the area of the wound at 12 h in relation to the initial total area. Values are the mean ± SD of one representative experiment out of three with similar results. One-way ANOVA and the Bonferroni's post-test was used to compare data; ∗*p* < 0.0 and ∗∗*p* < 0.01. EC, endothelial cell; HUVEC, human umbilical vein endothelial cell.

We have recently shown that vp17s are biologically active on endothelial cells (ECs) (5, 32). To evaluate whether secreted vp17s were active on ECs, we performed a wound healing assay, by cocultivation of nucleofected HeLa cells and human umbilical vein endothelial cells (HUVECs). A Culture-Insert 2 Well was used to provide two reservoirs for culturing cells that were separated by a 500 μm thick wall. The HUVEC and nucleofected HeLa cells were seeded in the reservoirs and cultured until they attached and formed a monolayer. Removal of the silicone insert from the surface was resulting in two precisely defined cell patches, which were separated by a zone that is exactly the same width as the separation wall. Cell migration of HUVECs was calculated as a percentage of wound observed over a period of 12 h. The number of HUVECs in the wound area increased more quickly (Fig. 3*A*), and the wound area decreased more rapidly in refp17- and vp17s-treated cells as compared with control cells (mock). As shown in Figure 3*B*, HUVECs cocultivated with mock HeLa cells reached approximately 47% sealing (range from 45 to 49%) after 12 h of culture, whereas, at the same time, HUVECs cocultivated with nucleofected HeLa cells expressing refp17 or its variants reached 80 to 90% sealing. This finding attests for the capability of viral proteins to promote EC migratory activity. Taken together, these data demonstrate that vp17s are secreted from infected cells in their biologically active form.

### Secreted p17s accumulate on cellular surface by binding to HSPGs

**Figure 4:**
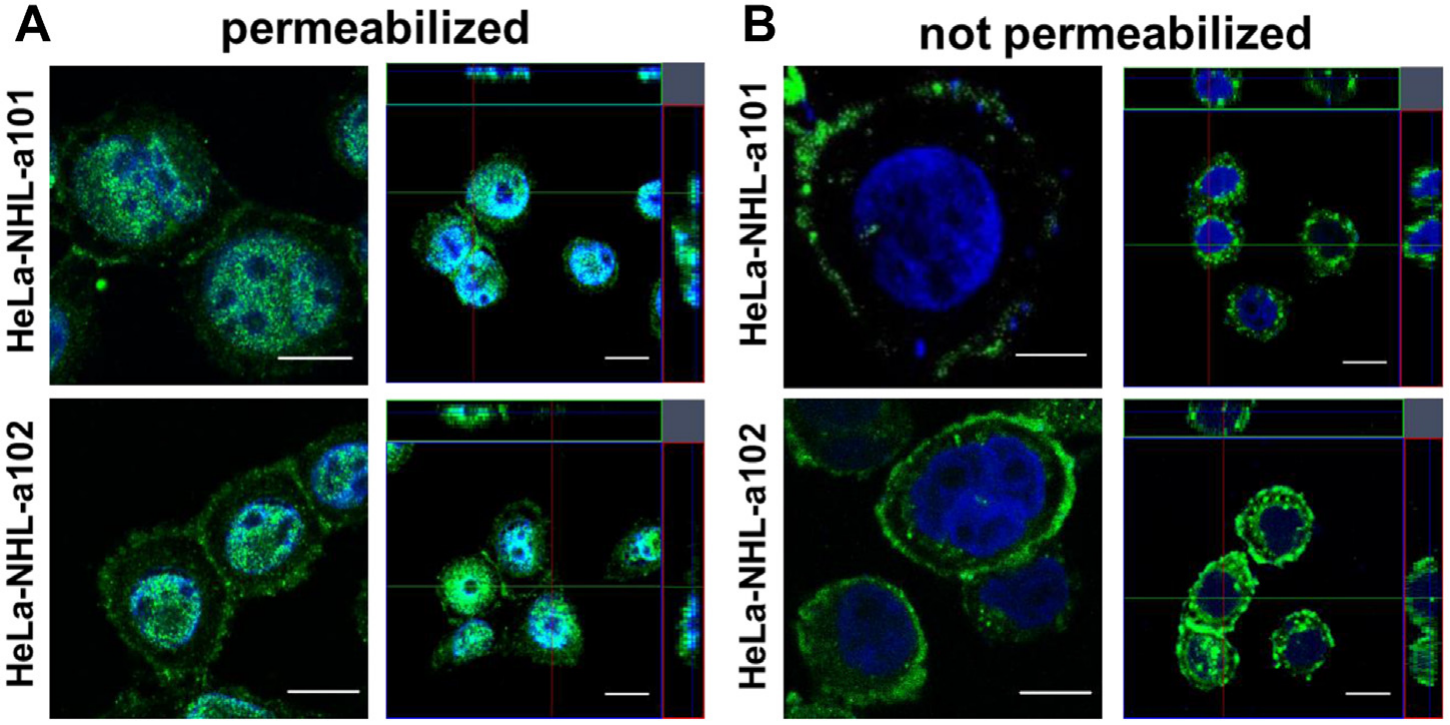
**Intracellular localization of vp17s in *Gag*-expressing cells.** HeLa cells were nucleofected with *Gag*-expressing plasmids carrying NHL-a101 (HeLa-NHL-a101) or NHL-a102 (HeLa-NHL-a102) and then cultured for 24 h at 37 °C. Cells were (*A*) permeabilized or (*B*) not permeabilized as described in the Experimental procedures section. Cells were then stained with anti-p17 mAb MBS-3 followed by Alexa Fluor 488–conjugated antimouse IgG and 4′,6-diamidino-2-phenylindole. Analysis was performed by confocal fluorescence microscopy. Images display mAb MBS-3 signals in *green* and cell nuclei in *blue*. z-Stack sections and orthogonal z reconstitution are also shown. The scale bar represents 10 μM. vp17, HIV-1 matrix protein p17 variant.

**Figure 5:**
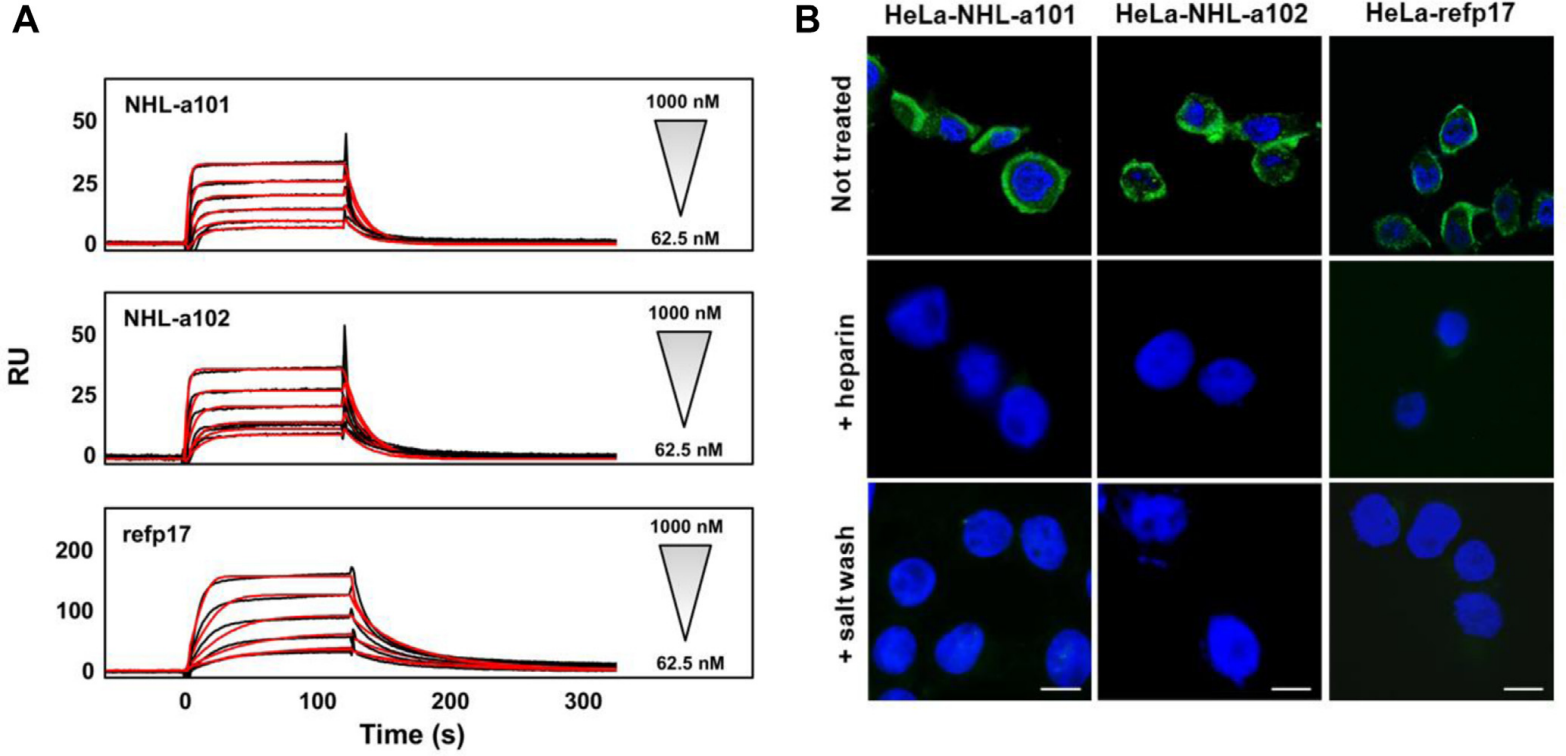
**P17s associate with cellular HSPGs.***A*, binding of p17s to heparin analyzed by SPR. Refp17 and its variants were injected on heparin sensor chip surface. All the proteins were tested at increasing concentrations (from 62.5 to 1000 nM). Affinity values of interaction were determined by BIAevaluation software. *B*, HeLa cells were nucleofected with *Gag*-expressing plasmids carrying refp17 (HeLa-refp17), NHL-a101 (HeLa-NHL-a101), or NHL-a102 (HeLa-NHL-a102) and then either incubated for 24 h in the presence of soluble heparin (10 μg/ml) or washed with PBS supplemented with 2 M NaCl (salt wash). Not permeabilized cells were then stained with anti-p17 mAb MBS-3 followed by Alexa Fluor 488–conjugated antimouse IgG and 4′,6-diamidino-2-phenylindole. Analysis was performed by fluorescence microscopy. Images display mAb MBS-3 signals in *green* and cell nuclei in *blue*. The scale bar represents 5 μm. HSPG, heparan sulfate proteoglycan; SPR, surface plasmon resonance.

Recently, we demonstrated that refp17 can be detected inside the cell as a punctate staining at the plasma membrane (26). To evaluate the cellular localization of vp17s, we carried out an indirect immunofluorescence assay and confocal microscopy analysis. HeLa cells were nucleofected with *Gag*-expressing plasmids as described previously (HeLa-refp17, HeLa-NHL-a101, and HeLa-NHL-a102), then fixed, and permeabilized or not before staining with the anti-p17 mAb MBS-3. To avoid mAb MBS-3 internalization, live and not permeabilized cells were maintained on ice during the staining step. Representative confocal images of permeabilized cells show that vp17s are localized at the plasma membrane as a punctate staining (Fig. 4*A*), as for refp17. On the contrary, in not permeabilized HeLa cells, both vp17s are confined on cell surface only (Fig. 4*B*). Previously, we have shown that refp17 is able to bind the HSPGs because of the presence of two distinct heparin-binding domains (33). To scrutinize the binding of vp17s to HSPGs, we performed a surface plasmon resonance (SPR) assay with heparin bound on the sensor chip. As shown in Figure 5*A*, both vp17s were found to bind heparin in a dose-dependent manner. The sensorgram overlay shown in Figure 5*A* allowed the calculation of association (*K*_on_) and dissociation (*K*_off_) rates and of *K*_d_ (as *K*_off_/*K*_on_ ratio) (Table 1). Based on this evidence, we hypothesize that HSPGs could be involved in vp17s binding on the cellular surface. Then, live and not permeabilized nucleofected HeLa cells were maintained for 24 h in the presence or absence of soluble heparin (10 μg/ml) or washed with 2 M NaCl (salt wash) before staining for indirect immunofluorescence assay. As shown in Figure 5*B*, not permeabilized cells treated with soluble heparin or salt washed, completely lost the expression of refp17 and vp17s on the cell surface, as compared with not treated cells. This finding suggests for the key role played by HSPGs in tethering refp17 and vp17s on cellular surface following their secretion.

**Table 1:** The association rate (*K*_on_) and dissociation rate (*K*_off_) are reported and the dissociation constant (*K*_*d*_) was derived from the *K*_off_/*K*_on_ ratio

Protein	Association rate (*K*_on_)	Dissociation rate (*K*_off_)	Dissociation constant (*K*_*d*_)
	1/Ms	1/s	M
NHL-a101	8.84E + 5	0.077	8.71E − 8
NHL-a102	8.41E + 5	0.059	7.01E − 8
refp17	2.62E + 5	0.148	5.64E − 8

### Secretion of vp17s takes place directly at the plasma membrane level following binding to PI(4,5)P_2_

We previously showed that *Gag* can be processed in the absence of HIV-1 protease being p17 digested from Pr55^*Gag*^ by cellular intramembrane aspartyl proteases following its binding to PI(4,5)P_2_, and that this process represents an essential step in promoting refp17 secretion (25). To evaluate if secretion of vp17s follows the same mechanism observed with refp17 in the absence of HIV-1 protease, we treated nucleofected HeLa-NHL-a101 and HeLa-NHL-a102 with soluble neomycin sulfate, a polycationic aminoglycoside antibiotic that binds to PI(4,5)P_2_ impairing the binding of PI(4,5)P_2_ ligands by steric hindrance (34), and pepstatin A, an inhibitor of cellular aspartyl proteases (35).

**Figure 6:**
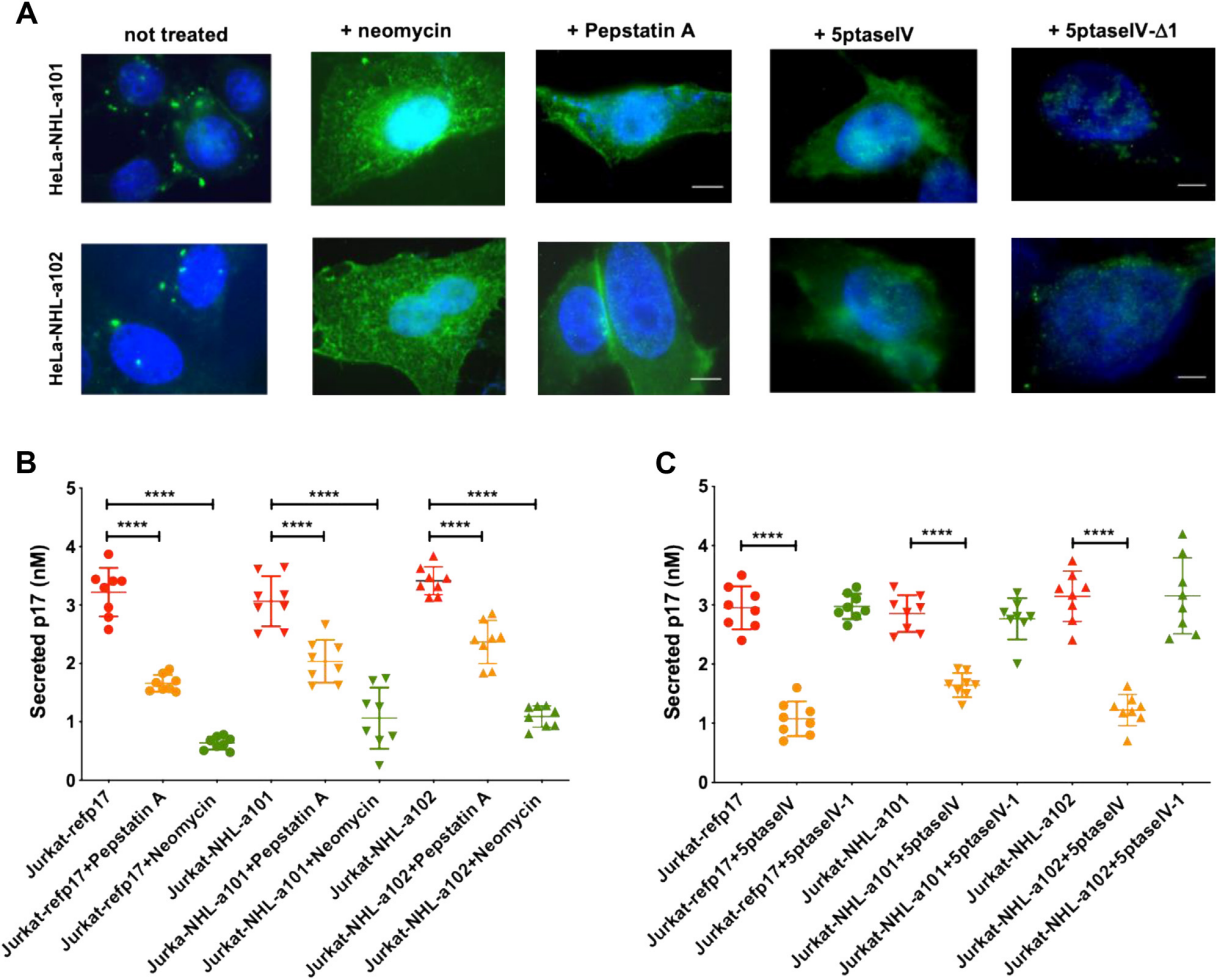
**Secretion of vp17s requires PI(4,5)P**_**2**_**binding at the plasma membrane level.***A*, HeLa cells were nucleofected with *Gag*-expressing plasmids carrying NHL-a101 (HeLa-NHL-a101) or NHL-a102 (HeLa-NHL-a102) and treated with pepstatin A (10 μM) or neomycin (500 μM). In some experiments, HeLa-NHL-a101 and HeLa-NHL-a102 were conucleofected with 5ptaseIV or 5ptaseIV-Δ1 mutant expression plasmids. Twenty-four hours later, permeabilized cells were stained with anti-p17 mAb MBS-3 followed by Alexa Fluor 488–conjugated antimouse IgG and 4′,6-diamidino-2-phenylindole. Analysis was performed by fluorescence microscopy. Images display mAb MBS-3 signals in *green* and cell nuclei in *blue*. The scale bar represents 5 μm. *B*, Jurkat cells were nucleofected with the pNL4-3 vector expressing refp17, NHL-a101, or NHL-a102 (Jurkat-refp17, Jurkat-NHL-a101, and Jurkat-NHL-a102, respectively) and treated or not with pepstatin A (10 μM) or neomycin (500 μM). *C*, Jurkat-refp17, Jurkat-NHL-a101, or Jurkat-NHL-a102 was conucleofected with 5ptaseIV or 5ptaseIV-Δ1 mutant expression plasmids. The evaluation of released vp17s was performed by cellular ELISA. Scatter plot was generated using the means of eight experiments. One-way ANOVA and the Bonferroni's post-test was used to compare data; ∗∗∗∗*p* < 0.0001. PI(4,5)P_2_, phosphatidylinositol-(4,5)-bisphosphate; vp17, HIV-1 matrix protein p17 variant.

As shown in Figure 6*A*, permeabilized HeLa-NHL-a101 and HeLa-NHL-a102 showed vp17s expression as a punctate staining at the plasma membrane. On the contrary, neomycin-treated cells showed a diffuse p17 expression in the cytoplasm. At the same time, pepstatin A treatment showed a dispersed vp17s expression within the cell cytoplasm and more pronounced at the plasma membrane (Fig. 6*A*). This result suggests that neomycin and pepstatin A favor intracellular vp17s accumulation by interfering with its enzymatic cleavage from Pr55^*Gag*^, as already observed in refp17-expressing cells (26). To further prove that vp17s secretion mechanism depends on the binding of viral proteins to PI(4,5)P_2_ at the cellular plasma membrane, we examined p17 secretion in cells depleted for PI(4,5)P_2_ by overexpression of 5ptaseIV (36). As shown in Figure 6*A*, HeLa-NHL-a101 and HeLa-NHL-a102 overexpressing 5ptaseIV displayed a diffuse distribution of vp17s in the cytoplasm. In contrast, HeLa-NHL-a101 and HeLa-NHL-a102 overexpressing the mutant form of 5ptseIV (5ptaseIV-Δ1) showed a punctate staining at the plasma membrane. These results are in agreement with previous data obtained with HeLa-refp17 (26).

In order to understand if refp17 and vp17s are secreted following their binding to PI(4,5)P_2_ during HIV-1 replication, we analyzed the effect of neomycin and pepstatin A on secretion of viral proteins following nucleofection with plasmids expressing the wildtype HIV-1 or HIV-1 mutants carrying NHL-a101 or NHL-a102 vp17s. As shown in Figure 6*B*, neomycin and pepstatin A were found to significantly inhibit the secretion of both NHL-a101 and NHL-a102 vp17s as well as of refp17, compared with untreated cells, thus confirming that the inhibition of Pr55^*Gag*^ binding to PI(4,5)P_2_ or its enzymatic cleavage prevents the release of vp17s. We then examined the impact of PI(4,5)P_2_ depletion on vp17s secretion by cellular ELISA. As shown in Figure 6*C*, secretion of refp17 and vp17s was strongly impaired in overexpressing 5ptaseIV. At the same time, Jurkat-refp17, Jurkat-NHL-a101, and Jurkat-NHL-a102 overexpressing 5ptaseIV-Δ1 released p17 at concentrations not significantly different from those detected in the supernatant of control cells, thus confirming the key role played by PI(4,5)P_2_ in vp17s secretion.

## Discussion

In light of the emerging role of the microenvironment in promoting and sustaining the growth and survival of tumor cells (37, 38), the possibility that HIV-1–encoded proteins derived from infected cells and endowed with peculiar biologic properties contribute to lymphomagenesis was entertained. A previous study has shown that a vp17 increases cell proliferation of Epstein–Barr virus–infected primary human B cells (39). Moreover, a more recent analysis showed a potential role of vp17s in promoting lymphoma in HIV^+^ patients, thus providing a rational explanation for the paradox of persistent risk for lymphoma among patients despite the immune-restorative effects of cART. Indeed, biologically active vp17s capable of augmenting clonogenic proliferation of lymphoma cell lines were detected in lymphoma tissue of patients with NHL and mirrored in their plasma counterparts (6).

Refp17 is released in a biologically active form by *Gag*-expressing cells, and secretion of the viral protein takes place at the plasma membrane, following refp17 interaction with PI(4,5)P_2_. The release of refp17 occurs following its cleavage from Pr55^*Gag*^ by cellular aspartyl proteases (26). Here, we show for the first time that two different vp17s, characterized by amino acid insertion at position 117 to 118 or 125 to 126, are secreted following the same unconventional mechanism previously described for refp17. Moreover, we show that vp17s are secreted in a biologically active form by both infected and *Gag*-nucleofected cells, as attested by their ability to trigger B-cell proliferation and transformation and promote EC migration. This finding attests that secretion of vp17s can occur both in the presence or the absence of active viral proteases. In the latter case, secretion of vp17s was found to be promoted by cellular aspartyl proteases–operated cleavage and be depending on the vp17s binding to PI(4,5)P_2_. In particular, our data show that Pr55^*Gag*^ binding to PI(4,5)P_2_ on plasma membrane is an essential step in promoting vp17s cleavage and secretion and that reduction of cellular PI(4,5)P_2_ levels by 5ptaseIV overexpression strongly impaired vp17s secretion. Previous findings showed the critical role of specific amino acids included in the HBR in refp17–PI(4,5)P_2_ interaction and/or in stabilizing such interaction and consequently allowing refp17 secretion. In particular, mutation of Lys at position 30 and 32 was found to strongly affect Pr55^*Gag*^–PI(4,5)P_2_ interaction and refp17 secretion, whereas Trp mutation at position 36 completely abolished p17 secretion. This latter finding can be, at least in part, explained by the knowledge that the side chain of W36 interacts with the glycerol moiety and with the C4-acyl chain of PI(4,5)P_2_ (27). It is worth noting that W36 is a highly conserved amino acid and possibly evolutionarily relevant, given that it is maintained in its ancestor simian immunodeficiency virus matrix protein, whose extracellular biological activities have been recently found to resemble, for many aspects, those of the HIV-1 vp17s (15). The evidence that these critical amino acid residues are well preserved on the primary sequence of both NHL-a101 and NHL-a102 explain how such high conservation, which occurs for all p17 amino acid residues strictly involved in PI(4,5)P_2_ interaction, may be necessary for sites that interact with cellular constituents that do not undergo evolutionary changes on the timescale of HIV-1 replication. Clonogenic vp17s are destabilized as compared with refp17 (6, 16). This suggests that protein destabilization, induced by mutations (deletions and insertions included) not restricted to the C terminus of p17, may ensue a conformational change sufficient to endow the NH_2_-terminal region of the viral protein with B-cell growth-promoting activity, presumably through better protease-activated receptor 1 recruitment and activation. At the same time, conformational changes in the N-terminal region do not alter the PI(4,5)P_2_ interacting epitope, which is indispensable for HIV-1 envelope incorporation and particle assembly at the plasma membrane (29, 40).

It has been demonstrated that even during HIV-1 latency, cells can produce *Gag* polyproteins without supporting virus assembly and spreading of infection being sufficient for the presence of unspliced RNA to allow the nuclear export and translation of *Gag* (9). This knowledge is supported by the evidence that *Gag* can be processed in the absence of HIV-1 protease (26) and give rise to p17 proteins capable of being released, in a biologically active form, in the extracellular microenvironment. These findings, together with the recent evidence showing the persistence of defective proviruses in HIV-infected individuals during cART capable of producing HIV viral proteins (8), contribute to the evidence that vp17s can be released also during HIV-1 latency leading to lymphatic B-cell stimulation, growth, and transformation. Popovic *et al.* (4) previously demonstrated persistence of p17 but not viral RNA in lymph nodes of patients on cART who were virally suppressed, suggesting the presence of soluble p17 in these tissues. If so, the impact of vp17s on B-cell growth and transformation is likely to persist. Recent data have highlighted the capability of vp17s to also promote both angiogenesis and lymphangiogenesis (41, 42), which are essential in supporting proliferation and survival of lymphoma, as well as tumor cell dissemination (43). Altogether, these evidences corroborate the hypothesis that vp17s actively secreted may be stored within a variety of organs and tissues where they may likely function as promoter of B-cell growth and transformation. Derived from our data are fundamental insights into the molecular basis on the key role that vp17s may play in lymphomagenesis, calls for inhibiting the biological activity of vp17s. This may be achieved in the microenvironment, by inoculating neutralizing antibodies (17), or by using specific therapeutic vaccines (44) aimed to generate a strong and efficient neutralizing antibody response. But it may be possibly realized also at the intracellular level, by avoiding Pr55^*Gag*^–PI(4,5)P_2_ interaction through anti-p17 antibody gene transduction (45) or small-molecule inhibitors able to engage the PI(4,5)P_2_ binding pocket of the viral protein (46).

In this study we show, for the first time, that both refp17 and vp17s are secreted in the extracellular environment but are also tethered to the cell surface through HSPGs. Amount of viral proteins associated with HSPGs, in addition to the free protein in the plasma, could represent a way to interact with specific receptors expressed on other—not necessarily infected—cells, and possibly cause bystander activation and dysfunction. In this view, it is likely that cell–cell contact can be implicated in promoting the highly efficient direct passage of the viral protein from one cell to another, acting as a virological synapse. Manipulation of the synapse may prevent an effective immune response to the virus, similarly to previous observation of evasion strategies by which Nef prevents recognition of infected cells by cytotoxic T lymphocytes and with other effects of the viral protein on cytokine secretion by macrophages and dendritic cells (47). On the other hand, the finding of p17 tethering on the surface of HIV-1 reservoirs may offer new opportunities to promote the development of therapeutic strategies aimed to HIV-1 eradication. Up to date, one of the most prominent approaches to achieve HIV-1 cure with the aim of reducing the viral reservoir is “shock and kill,” which uses latency reversing agents to induce viral gene expression and productive infection in latently infected cells, exposing those cells to immune clearance or to the viral cytopathic effect (48). Expression of p17 on the surface of infected cells in the absence of active virus replication may ultimately represent a target for immune-mediated clearing (“kill”), thus allowing elimination of viral reservoirs even in the absence of HIV-1 latency reversal. This hypothesis needs to be validated in suitable *in vitro* and *in vivo* models of HIV-1 latency.

## Experimental procedures

### Expression vectors

pNL4-3 vector, containing a full infectious provirus, HIV-1_NL4-3_, was a kind gift of Dr Anna Cereseto (University of Trento, Italy). Tat-independent AG49CMVIL15-RTEm26CTE vector was kindly provided by Dr Barbara Felber (National Cancer Institute, Frederick). The *Gag*-expressing plasmid AG49CMV*Gag*-RTEm26CTE was generated as previously described (26). The plasmids carrying vp17s, namely NHL-a101 and NHL-a102, were generated by substituting the refp17 within the *Gag* gene, with vp17s synthetic genes (Integrated DNA Technologies). Cells nucleofected without plasmid were used as negative control (mock). The 5ptaseIV expression plasmid, pcDNA4TO/Myc5ptaseIV, was a gift from P. Majerus (Washington University School of Medicine, St Louis, MO). The 5ptaseIV mutant lacking the phosphatase signature domain (Δ1) was a gift from A. Ono (University of Michigan Medical School, Ann Arbor, MI).

### Recombinant proteins and mAbs to p17

Monomeric HIV-1 matrix protein p17 and its variants were produced and purified as previously described (16). The absence of endotoxin contamination (<0.25 endotoxin units/ml) in protein preparations was assessed by Limulus amoebocyte assay (Associates of Cape Cod). mAbs to p17 (neutralizing mAbs: MBS-3 and MBS-34; not neutralizing mAbs: MBS-15 MBS-55) were produced in our laboratory (5, 49).

### Cells

HeLa, Jurkat, and Raji cell lines (American Type Culture Collection) were maintained in RPMI1640 (Merck) supplemented with 10% fetal bovine serum (FBS) (Merck), sodium pyruvate, and l-glutamine (2 mM). HUVECs have been previously developed and characterized (5). Cells were cultured in endothelial cell growth medium (EGM) supplemented with 10% FBS. All the experiments were carried out with cells at passages 2 to 6. Cells were maintained at 37 °C in a humidified atmosphere of 5% CO_2_.

### vp17s secretion assay

Jurkat cells were nucleofected using the Amaxa Nucleofector II device (Lonza) and the Ingenio Electroporation Universal kit (Mirus). Twenty-four hours after nucleofection, cells were washed with RPMI1640, resuspended in complete medium, and then transferred to an ELISA MaxiSorp Nunc plate (7.5 × 10^5^ cells/well, in triplicate) previously coated overnight at 4 °C with the anti-p17 mAb MBS-15 at 10 μg/ml in PBS. After 24 h of incubation, cells were removed and biotinylated anti-p17 mAb (MBS-55 or MBS-34 [1 μg/ml] for NHL-a101 and NHL-a102, respectively) was added to the wells for 1 h at 37 °C. Detection of immunocomplexes was performed using a peroxidase-labeled streptavidin (BD Biosciences) and the 3,3,5,5-tetramethylbenzidine (Merck) as chromogenic substrate. Standard curves for p17 and its variants were generated by serial dilution of the recombinant proteins (from 0.125 to 18 nM), and the amount of secreted p17s was calculated by linear interpolation. In some experiments, nucleofected cells were treated with neomycin (500 μM) [as PI(4,5)P2 inhibitor] (Life Technologies) and pepstatin A (10 μM) (as inhibitor of aspartyl proteases) (Merck). When reported, Jurkat cells were conucleofected with the 5ptaseIV expression plasmid pcDNA4TO/Myc5ptaseIV66 or with the mutant expression plasmid pcDNA4TO/Myc Δ15ptaseIV (36, 50).

### B-cell colony formation assay

HeLa cells were nucleofected with *Gag* plasmid expressing refp17, NHL-a101, or NHL-a102. Twenty-four hours later, cell supernatants were collected and centrifugated to remove cell debris. The clarified medium was used to perform the colony formation assay (17). Raji cells resuspended in conditioned supernatants were seeded by manual pipetting into a 96-well plate at a dilution of 0.5 cells/well. When reported, conditioned supernatants were preincubated for 30 min at 37 °C with 1 μg/ml of unrelated Ctrl mAb or p17 neutralizing mAb MBS-3. After 8 days of incubation, the culture plates were analyzed for single colony formation.

### Wound healing assay

The Culture-Insert 2 Well (Ibidi GmbH) was used to perform the assay. In the first well, HUVEC cells were plated (1 × 10^5^ cells/well) in EGM containing 10% FBS, and confluent cell monolayers were starved for 24 h by replacing medium with endothelial basal medium containing 0.5% FBS. In the second well, HeLa cells (1 × 10^5^ cells/well) were plated after nucleofection with *Gag* plasmid expressing refp17, NHL-a101, or NHL-a102. Twenty-four hours later, the Culture-Insert 2 Well were gently removed with sterile tweezers creating a physical gap within the two cell monolayers. Cell layers were washed with PBS to remove cell debris and not attached cells. The μDish was filled with cell medium (EGM supplemented with 10% FBS) applying the recommended volume of 2 ml. HUVEC migration in response to refp17 or variant proteins secreted by nucleofected HeLa cells was evaluated at different time points using an inverted microscope (DM-IRB microscope system; Leica). HUVECs migrating into the wounded area, or protruding from the border of the wound, were photographed using a charge-coupled device camera (Hitachi, Inc) connected to a computer. The areas of the wound were calculated by using the ImageJ software (National Institutes of Health).

### Immunofluorescence analysis

HeLa cells were nucleofected with AG49CMV*Gag*-RTEm26CTE plasmid expressing refp17, NHL-a101, or NHL-a102 (HeLa-ref, HeLa-NHL-a101, and HeLa-NHL-a102, respectively), cultured for 24 h in complete medium, and then stained for immunofluorescence as follows: fixed with 3% paraformaldehyde/2% sucrose in PBS for 30 min, permeabilized with 0.5% Triton, and saturated with 1% bovine serum albumin (BSA) in PBS. Then, the cells were incubated for 1 h with the anti-p17 mAb MBS-3 followed by Alexa Fluor 488–conjugated antimouse IgG (Molecular Probes) at room temperature. When indicated, not fixed cells were saturated with 1% BSA in PBS, incubated for 1 h with the anti-p17 mAb MBS-3 followed by Alexa Fluor 488–conjugated antimouse IgG on ice and then fixed as aforementioned. Nuclei were counterstained with 4′,6-diamidino,2-phenylindole (DAPI; Sigma). When reported, nucleofected HeLa cells were also treated with neomycin (500 μM) [as PI(4,5)P2 inhibitor] (Life Technologies) or with the specific cellular aspartyl protease inhibitor pepstatin A (10 μM) (Merck). In some experiments, HeLa cells were conucleofected with the 5ptaseIV expression plasmid pcDNA4TO/Myc5ptaseIV66 or with the mutant expression plasmid pcDNA4TO/Myc Δ15ptaseIV. In selected experiments, cells were treated with 10 μg/ml of the conventional unmodified heparin (13.6 kDa) (Laboratori Derivati Organici S.p.A.) or salt washed with 2 M NaCl (Sigma). Fluorescence was recorded with a Nikon DXM 1200 digital camera system (Nikon) coupled to the Eclipse E1000 fluorescence microscope (Nikon) and the ACT-1 control software (Nikon).

### Confocal analysis

HeLa cells were nucleofected with AG49CMVGag-RTEm26CTE plasmid expressing NHL-a101 or NHL-a102 (HeLa-NHL-a101 and HeLa-NHL-a102, respectively), cultured for 24 h in complete medium, and then stained for confocal microscopy analysis as follows: fixed with 3% paraformaldehyde/2% sucrose in PBS for 30 min, permeabilized with 0.5% Triton, and saturated with 1% BSA in PBS. Then, the cells were incubated for 1 h with the anti-p17 mAb MBS-3 followed by Alexa Fluor 488–conjugated antimouse IgG (Molecular Probes) at room temperature. When indicated, not fixed cells were saturated with 1% BSA in PBS, incubated for 1 h with the anti-p17 mAb MBS-3 followed by Alexa Fluor 488–conjugated antimouse IgG on ice and then fixed as aforementioned. Nuclei were counterstained with DAPI (Sigma). Cells were analyzed using a Zeiss LSM510 Meta confocal microscope equipped with a Plan-Apochromat 63×/1.4 numerical aperture oil objective. The excitation sources were an argon ion and 405-nm diode lasers for Alexa Fluor 488 and DAPI, respectively. Alexa Fluor 488 was acquired using a LP 505 filter, whereas DAPI was acquired using a LP 420 combined with an NFT 490 beam splitter. Single images and orthogonal projection from z-stack sections were obtained through Zen Black 2 software (Zeiss).

### SPR experiments

SPR measurements were conducted on a Biacore X100 (GE Healthcare) at 25 °C. SPR was used to characterize the binding of HIV-1 p17s to heparin immobilized on a sensor chip. Biotinylated heparin was immobilized onto a SA sensor chip containing preimmobilized streptavidin, allowing the immobilization of 115 resonance units, equal to 8.5 fmol/mm^2^ of heparin. A sensor chip precoated with streptavidin alone was used to evaluate nonspecific binding and for blank subtraction (51). Increasing concentrations of p17 or its variants in 10 mM Hepes, pH 7.4 containing 150 mM NaCl, 3 mM EDTA, and 0.005% surfactant P20 (HBS-EP) were injected over the heparin or streptavidin surfaces for 4 min and then washed until dissociation. After each run, the sensor chip was regenerated by injection of 2 M NaCl in HBS-EP. Kinetic parameters were calculated from the sensorgram overlays by using the nonlinear fitting single-site model software package BIAevaluation (version 3.2 [GE Healthcare]). Only sensorgrams whose fitting gave χ^2^ values close to 10 were used (52).

### Statistical analysis

Data obtained from multiple independent experiments are expressed as the mean ± SD. Data were analyzed for statistical significance using the one-way ANOVA. Bonferroni's post-test was used to compare data. Differences were considered significant at *p* < 0.05. Statistical tests were performed using GraphPad Prism 8 software (GraphPad Software, Inc).

## Data availability

All data have been included within the article.

## Conflict of interest

The authors declare that they have no conflicts of interest with the contents of this article.
